# β-Alanine supplementation increased physical performance and improved executive function following endurance exercise in middle aged individuals

**DOI:** 10.1186/s12970-018-0238-7

**Published:** 2018-07-11

**Authors:** Taylor Furst, Alyssa Massaro, Courtney Miller, Brian T. Williams, Zach M. LaMacchia, Peter J. Horvath

**Affiliations:** 1 Jacobs School of Medicine and Biomedical Sciences, University at Buffalo, Buffalo, USA; 2 Department of Exercise and Nutrition Sciences, University at Buffalo, Buffalo, USA; 3 Department of Physiology and Biophysics, University at Buffalo, Buffalo, USA; 4 Department of Pathology and Anatomical Sciences, University at Buffalo, Buffalo, USA

**Keywords:** β-Alanine, Carnosine, Executive function, Exercise, Stroop test, Time-to-exhaustion, Lactate

## Abstract

**Background:**

Sarcopenia, a reduction in muscle mass and function seen in aging populations, may be countered by improving systemic carnosine stores via beta-Alanine (β-alanine) supplementation. Increasing systemic carnosine levels may result in enhanced anti-oxidant, neuro-protective and pH buffering capabilities. This enhancement should result in improved exercise capacity and executive function.

**Methods:**

Twelve healthy adults (average age = 60.5 ± 8.6 yrs, weight = 81.5 ± 12.6 kg) were randomized and given either 2.4 g/d of β-alanine (BA) or Placebo (PL) for 28 days. Exercise capacity was tested via bouts on a cycle ergometer at 70% VO_2_ peak. Executive function was measured by Stroop Tests 5 min before exercise (T1), immediately before exercise (T2), immediately following fatigue (T3), and 5 min after fatigue (T4). Lactate measures were taken pre/post exercise. Heart rate, Rating of Perceived Exertion (RPE) and VO_2_ were recorded throughout exercise testing.

**Results:**

PRE average time-to-exhaustion (TTE) for the PL and BA group were not significantly different (Mean ± SD; 9.4 ± 1.4mins vs 11.1 ± 2.4mins, respectively, *P* = 0.7). POST BA supplemented subjects cycled significantly longer than PRE (14.6 ± 3.8mins vs 11.1 ± 2.4mins, respectively, *P* = 0.04) while those given PL did not (8.7 ± 2.4mins vs 9.4 ± 1.4mins, respectively, *P* = 0.7). PL subjects were slower in completing the Stroop test POST at T4 compared to T3 (T3 = − 13.3 ± 8.6% vs T4 = 2.1 ± 8.3%, *P* = 0.04), while the BA group (T3 = − 9.2 ± 6.4% vs T4 = − 2.5 ± 3.5%, *P* = 0.5) was not. POST lactate production expressed a trend when comparing treatments, as the BA group produced 2.4 ± 2.6 mmol/L more lactate than the PL group (*P* = 0.06). Within group lactate production for BA (*P* = 0.4) and PL (*P* = 0.5), RPE (*P* = 0.9) and heart rate (*P* = 0.7) did not differ with supplementation.

**Conclusion:**

BA supplementation increased exercise capacity and eliminated endurance exercise induced declines in executive function seen after recovery. Increased POST TTE coupled with similar PRE vs POST lactate production indicates an improvement in the ability of BA to extend exercise durations. Furthermore, by countering endurance exercise’s accompanying deficits in executive function, the aging population can maintain benefits from exercise with improved safety.

## Background

Aging is often associated with a reduction in one’s ability to exercise. A common causative factor is age-related deterioration, known as sarcopenia, and changes in viable skeletal muscle [1]. It has been previously shown that there is a direct relationship between sarcopenia onset and depleted systemic carnosine [2–5]. Carnosine is a dipeptide synthesized by carnosine synthetase in the presence of β-Alanine and L-Histidine [4, 6–10] predominantly found within skeletal muscle. It functions to improve myofiber contractility via enhancing sarcomere sensitivity to calcium, as well as to maintain pH homeostasis [4, 6, 8, 10–15]. By acting as a pH buffer, carnosine allows for larger accumulation of lactate during exercise by delaying the associated acidification of systemic pH known to have negative effects on exercise performance and cognition [2, 6, 12]. As carnosine concentrations become depleted as a result of sarcopenia, its ability to buffer pH becomes limited manifesting a quicker onset of acidosis [2, 3].

Systemic carnosine levels have been successfully elevated by supplementing β-Alanine, a non-essential amino acid and rate limiting factor in carnosine synthesis [8, 16], at 3.2 g/day and 6.4 g/day [10, 17]. Additionally, β-Alanine induced increases in systemic carnosine are sustained over a range of 4 months following supplementation [14]. Studies have been investigated β-Alanine supplementation in young adults in an effort to increase exercise performance via multiple dosing strategies and exercise modalities. Hill et al. [12] showed that increasing the dose of β-Alanine from 4.0 g/day to 6.4 g/day over 4 weeks improves total work done by high intensity, college aged cyclists. Multiple other studies have also found similar results with varying dosage strategies. For example, Hoffman *et al* [9] found that 6.0 g of β-Alanine for 28 days improved tactical performance and jumping ability, but not serial subtraction test time in soldiers. 6.4 g/day was found to improve upper-body Wingate bouts [18].

The improvements in exercise capacity seen in young adults led to investigation involving β-Alanine supplementation in aging adults to combat symptoms of sarcopenia [2, 4]. Studies of aging populations have used a variety of dosage schemes such as 1.6 g/day, 2.4 g/day and 3.2 g/day for a range of 28 to 84 days [2–4]. Despite the positive results seen in younger populations, only del Favero et al. [2] has directly tested muscle carnosine or endurance cycling ability in aging subjects. They found an 85.4% increase in gastrocnemius carnosine levels as well as improved endurance exercise capacity following 12 weeks of 3.2 g/day of β-Alanine supplementation [2]. Different variations of dosages and length of supplementation within aging populations have, however, shown significant increases in cycling ability without directly measuring intramuscular carnosine levels [3, 4].

Furthermore, exercise has shown to affect executive functioning, such as decision making and short term memory [19]. Interestingly, researchers have shown that carnosine also accumulates in the central nervous system, specifically the cerebral cortex [13, 14]. In cerebral tissue, carnosine acts as an anti-oxidant with neuro-protective properties [7, 9, 13, 20]. Hoffman et al. [21] used rats to show that 30 day β-Alanine supplementation increases carnosine concentrations in the cerebral cortex, hippocampus, amygdala, hypothalamus and thalamus when exposed to stress. In humans, however, a 28 day β-Alanine supplementation showed improved physical fitness performance in military personnel, yet provided only minor improvements in decision making and reaction time [9]. Further, a recently published study was also unable to demonstrate improved executive function when testing at time points immediately prior to and following exercise [22].

The present study was undertaken to further investigate the effect of β-Alanine supplementation on exercise endurance and executive function in a middle aged human population. Our primary outcome was exercise performance measured as time-to-exhaustion (TTE). Our secondary outcome was Stroop Test derived indices of executive function. We hypothesize that β-Alanine supplementation would (a) improve exercise performance and (b) attenuate the decline in post exercise executive function.

## Methods

### Subjects

Twelve subjects (eight men, four women) were recruited from the Buffalo, New York area. All subjects were over the age of 50 years and postmenopausal. Though prescription medications were not considered as exclusion criteria, subjects were asked to report any changes made during their time enrolled in the study. Exclusion criteria for subjects were as follows: individuals following rigorous exercise plans, individuals using supplements within 6 months of their participation, color blindness to ensure the ability to perform Stroop tests, disabling pain while riding a bicycle, smoking individuals or those who have smoked within 6 months of their participation and failure to meet cardiovascular low risk criteria. Before testing was conducted, all subjects underwent an exercise-screening test to further ensure ability to perform the bike test and assess exercise capacity. Body composition was determined using a Bodpod (Cosmed, Chicago, IL). During the screening visit, demographic data were collected for all subjects (Table 1). The State University of New York University at Buffalo’s Institutional Review Board for Human Subjects approved all procedures conducted prior to the start of subject recruitment (IRB: 699720).

**Table 1:** Subject demographics

Subject Demographics (*n* = 12)		
Demographic	Mean ± SD	Range (low, high)
Age (yrs)	60.5 ± 8.6	51, 74
Height (m)	1.7 ± 0.1	1.7, 1.9
Weight (kg)	81.5 ± 12.6	52.2, 95.3
Body Fat Percent (%)	22.6 ± 10.6	18.7, 44.5
Fat Free Mass (kg)	67.7 ± 14.5	51.1, 98.5
Body Mass Index (kg/m^2^)	27.5 ± 3.3	20.4, 32.4
70% Peak Watts (watts)	110.4 ± 31.8	65, 180

Table 1. All subjects were non-smoking, middle age individuals with no formal exercise training regimens or moderate-high health risks.

### Experimental design

The study consisted of three visits, a screening (visit 1), pre-supplementation (visit 2), and post-supplementation (visit 3), and utilized a double blinded, placebo-controlled, parallel arm experimental design. Executive function and physical assessments were performed PRE and POST, which included the Stroop test, a TTE trial via cycle ergometer and lactate measures both prior to and following exercise. During all exercise bouts, VO_2_ was continuously measured via a Vacumed Metabolic Cart (Vacumed, Ventura, CA). Heart rate (HR) and Rate of Perceived Exertion (RPE) were recorded every 2 min during all exercise tests. All subjects were instructed to arrive to each visit after a 3 h fast and to refrain from strenuous activity prior to this. Subjects were also instructed to maintain their current activity level and diet throughout the course of the study. A 24-h diet recall was performed prior to visit 2 and visit 3 to ensure subject’s ingestion would not alter performance. Each subject’s visits took place at the same time of day. Serum blood was drawn and stored at − 70 °C PRE and POST for future study.

### Supplementation

Supplementation was modeled after a similar study conducted by Stout et al. [4] Immediately following the PRE visit, subjects were randomized into one of two treatments options, β-Alanine (BA) or Placebo (PL). The BA group (*n* = 7) was given 2.4 g of β-Alanine per day, while the Placebo group (*n* = 5) was given microcrystalline cellulose. Both forms of treatment were administered in identical clear gelatin capsules. Each capsule contained 800 mg of either BA or PL. Daily supplementation within ageing subjects is commonly separated into multiple, 800 mg doses per day to avoid BA induced paresthesia, which is a pins-and-needles/prickly sensation. This is a normal dose-dependent response commonly felt on the skin of the face and extremities following the ingestion of large doses of BA and the resultant peak in BA plasma concentration [23]. This sensation has been reported to last for roughly 1 h following onset [23]. It is thought that dosing strategies designed to lower doses and, therefore, the extent of plasma concentration peaks can be a preventative measure [23]. Subjects were instructed to ingest three capsules per day, one with each meal, for 28 days. To ensure subject compliance, subjects were given a supplementation log and instructed to record the date and time that each capsule was taken. Based upon subject intake records, no subjects reported skipping supplementation. Self-compliance in supplementation was therefore sufficient. Also, by dosing 2.4 g of BA in 800 mg doses, there were no subject complaints of paresthesia, an important factor in patient comfort. Both β-Alanine and microcrystalline cellulose were purchased from Sigma-Aldrich, Co., 3050 Spruce St., St. Louis, MO.

### Exercise testing

Pre and POST exercise testing were performed on a cycle ergometer as a continuous non-graded submaximal open-ended bout at 70% VO_2_ peak, an estimated ventilatory threshold (VT), otherwise known as a TTE trial. To determine each subjects’ 70% VO_2_ peak, a continuous graded exercise test (GXT) was conducted during their screening visit, a variation of that conducted by Stout et al. [24] The screening GXT was conducted as follows: following a self-conducted warm-up, subjects began cycling at a workload of 50 watts (W) and a cadence of 70 rpm (rpm). Every 2 min, watts were increased by 25 W and subjects were instructed to exercise until voluntary fatigue. If cadence fell below 70 rpm (rpm) for greater than 10 s or the subject felt they could not continue, investigators ended the test. VO_2_ was recorded continuously throughout the bout. HR and RPE were recorded every 2 min. Two-minute averages were calculated for the subject’s VO_2_ throughout the course of their GXT and plotted on a graph as a function of watts (watts vs. VO_2_) using Microsoft Excel. Workload for the subsequent TTE trials that are conducted in the PRE and POST visits was determined as the workload in watts that correlated to 70% of the subject’s VO_2_ peak, based upon the formulated graph.

Procedure for the PRE and POST TTE bout at 70% VO_2_ peak were as follows: Subjects began to warm up for 5 min at a self-selected pace and a workload of 50 W. Immediately following the 5-min warm up, workload in watts was increased to their respective, predetermined 70% peak. Subjects were instructed to maintain a cadence of 70 rpm at their respective workload until voluntary fatigue. Time was not started until subjects have reached a cadence of 70 rpm at their respective workload. If cadence fell below 70 rpm for greater than 10 s, the test was ended. VO_2_ was recorded continuously. HR and RPE were recorded every 2 min. For all exercise tests, lactate was measured via a LactatePlus (Nova Biomedical, Waltham, MA) analyzer immediately before and immediately following exercise. Lactate production was then calculated as the difference between blood lactate concentrations prior to and following exercise. TTE bouts were designed to assess subject’s endurance exercise capacity.

### Executive function testing

Executive function throughout the study was examined via the Stroop test, a cognitive task designed to assess working memory and response inhibition. Emphasis was placed on measuring declines in executive function following endurance exercise. The Stroop test consisted of a series of colors listed as words in two vertical columns of ten. On the left, ten words were written in their corresponding color (i.e., “GREEN” written in GREEN ink), while on the right ten new words were written in a mismatched color (i.e., “GREEN” was written in RED ink). Prior to beginning each test, subjects were instructed to give their verbal responses as quickly and accurately as possible; subjects were also instructed prior to beginning each test that if a mistake was made, they shall correct themselves and move on. Within each test, two tasks were asked of each subject similar to a study conducted by Solis et al. [22] Each task was performed on a separate pass through the words. During the first pass, subjects performed the first task in which they were to read the word ignoring the color of the ink (i.e., “GREEN” written in RED ink; correct answer is GREEN). The second task was performed during the second pass and consisted of simply identifying the color of the ink (i.e., “GREEN” written in RED ink; correct answer is RED). Scoring was conducted as Stroop’s original “Basic score,” that consisted of simply the time to complete each task [25]. Each task was timed via a stopwatch. Accuracy was measured, however, not scored because the time taken by a subject for corrected errors contributed to the overall time to completion [26].

Four Stroop tests were given during each visit, 1 5 min before exercise (T1), one immediately before exercise (T2), one immediately following fatigue (T3) and finally 1 5 min after fatigue (T4). Four Stroop tests were given during each visit to minimize a learning effect in performance. During each visit, T1 was treated as a true test; however, it was used as a practice for subsequent trials to re-familiarize the subject to testing procedure and not included in data analysis. Percent change was calculated as a change from PRE to POST within each time point (i.e., percent change from PRE T3 to POST T3). The percent change within each time point was then used to analyze differences between time points.

### Statistical analysis

TTE data sets were analyzed using Two Way Measures Analysis of Variance (ANOVA), while physiological measures and Stroop test data was analyzed using T-tests. All data was analyzed with a P level of 0.05 set to determine significance. All analysis tests were run using SigmaPlot version 13.0 software (Systat Software, San Jose, CA). Weighted effect sizes were calculated using Cohen’s d similar to previous studies [27–29]. Effect sizes are presented based upon a scale (small = 0.2; medium = 0.5; large = 0.8) set according to Homack et al. [29] An estimated sample size of 13 was calculated utilizing data from del Favero et al [2], an α of 0.05 and 95% confidence intervals, with a R^2^ of 0.8. The current study analyzed data from a sample size of 12, which resulted in a power of 0.9. Data are presented as Mean ± SD throughout unless otherwise noted.

## Results

A total of 13 subjects completed the study (PL = 6, BA = 7). One subject from the PL group was not included in data analysis due to an outlier TTE of 54.4 min and failure to disclose intense, routine cycling training until after exercise tests were performed. One subject withdrew from the study prior to being assigned to a treatment group due to personal reasons. The total number of subjects included in the data set analyzed is 12.

### TTE

**Fig. 1:**
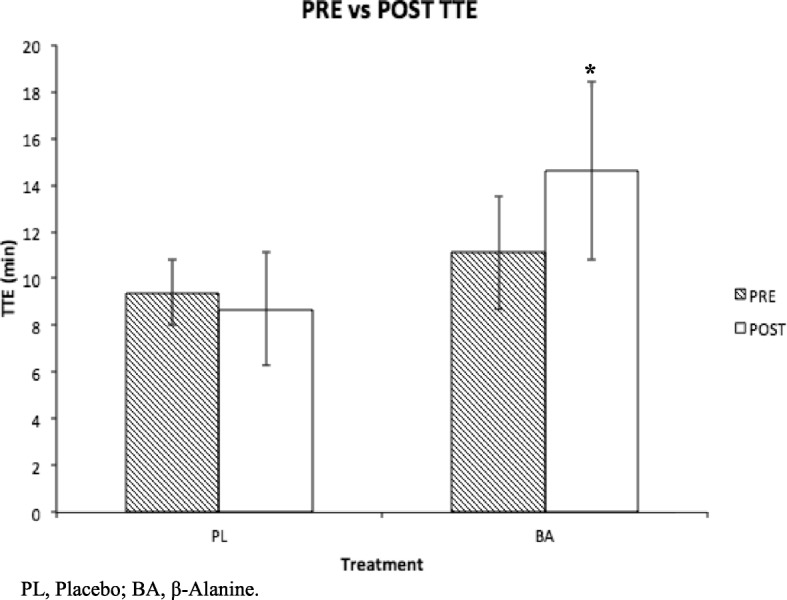
PRE vs POST Δ TTE. Results are represented as boxplots with * *P* < 0.05. Data represents mean ± SD. POST BA TTE was significantly longer than PRE (14.6 ± 3.8mins vs 11.1 ± 2.4mins, respectively, *P* = 0.04); PL TTE did not significantly change (PRE, 9.4 ± 1.4mins; POST, 8.7 ± 2.4mins, *P* = 0.7). PL, Placebo; BA, β-Alanine

Mean PRE TTE (10.3 ± 2.1 min) was similar among PL (*n* = 5) and BA (*n* = 7) groups (9.4 ± 1.4mins vs 11.1 ± 2.4 mins, respectively, *P* = 0.7). POST TTE was similar between treatments (*P* = 0.5; Effect Size = 0.02), time (*P* = 0.2; Effect Size = 0.003) and time-by-treatment (*P* = 0.09; Effect Size = .03). However, within treatment analysis indicated that POST TTE increased 24% from PRE TTE with BA supplementation (11.1 ± 2.4 min to 14.6 ± 3.8 min, *P* = 0.04; Effect Size = 0.4), while POST TTE was similar to PRE TTE after PL supplementation (9.4 ± 1.4 min to 8.7 ± 2.4 min, *P* = 0.7; Effect Size = 0.1). Fig 1.

### Physiological measures

**Fig. 2:**
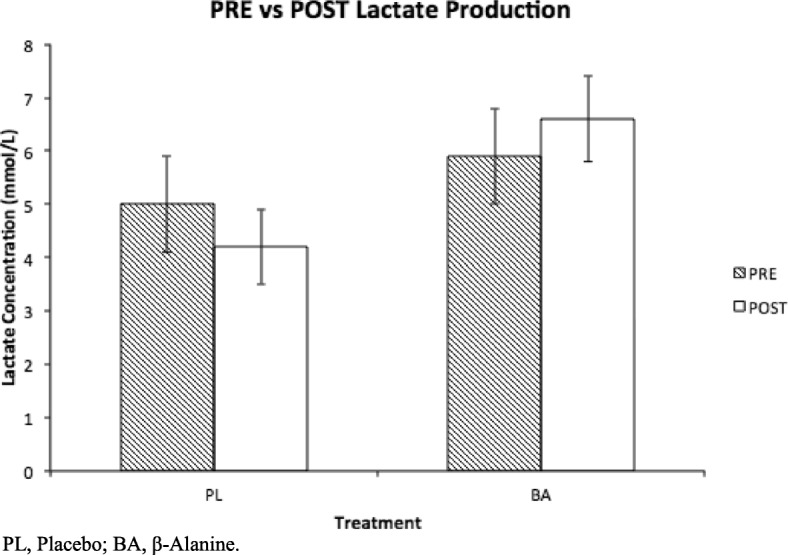
PRE vs POST lactate production. Results are represented as boxplots. Data represent mean ± SD. No change in lactate production within either group. When comparing treatments, POST lactate production expressed a trend as BA produced more lactate than PL (*P* = 0.06). PL, Placebo; BA, β-Alanine

Blood lactate concentration is commonly measured during clinical exercise testing as a marker of a “normal” physiological response to exercise representing exertion. Lactate production was measured as post-exercise subtracted from pre-exercise lactate concentration. PRE lactate production was similar among supplementation groups (BA = 5.9 ± 0.7 mmol/L vs PL = 5.0 ± 0.9 mmol/L, *P* = 0.5). POST lactate production expressed a trend as BA produced 2.4 ± 2.6 mmol/L more lactate than PL (BA = 6.6 ± 0.8 mmol/L vs PL = 4.2 ± 0.9 mmol/L, *P* = 0.06). Lactate production did not increase from PRE to POST within BA (*P* = 0.4) nor PL (*P* = 0.5). Fig 2. No differences were detected for average HR (*P* = 0.9) or RPE (*P* = 0.7) during exercise.

### Stroop tests

**Fig. 3:**
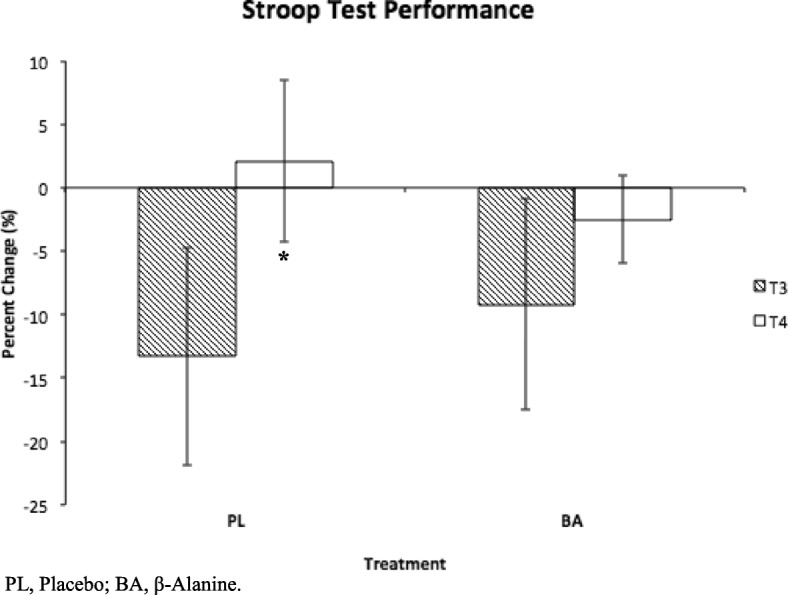
Stroop test performance. Percent change from PRE to POST time to perform the Stroop test task of identifying the colors. Positive change represents a decline in executive function. Data represent mean ± SD. Results are represented with * *P* < 0.05. BA mediated the decline in executive function following recovery from fatigue (T3 vs T4) seen within PL (*P* = 0.04). PL, Placebo; BA, β-Alanine

As a marker of executive function, subjects performed the Stroop test prior to and after each TTE test. A decline in executive function is represented as an increased time to complete the task. POST BA (T3 = − 9.2 ± 6.4% vs T4 = − 2.5 ± 3.5%, *P* = 0.5) did not have a decline in ability to identify colors as was seen with PL (T3 = − 13.3 ± 8.6% vs T4 = 2.1 ± 8.3%, *P* = 0.04). Fig 3. No significance was found when comparing T2 vs T3 or T4. No significance was found for time to complete the Stroop test task of reading the words at any time point. Based on Cohen’s d calculations, both the PL (1.9) and BA (1.5) groups corresponded to high effect size within Stroop test performance [28, 29].

## Discussion

This study shows that a 28-day BA supplementation in aging men and women increases endurance exercise performance and executive function. These findings specifically reinforce that BA supplementation correlates with improved exercise performance with potential secondary effects leading to improved muscle strength, decreased fall risk and improved cardiovascular health. More research is needed to further investigate the secondary effects of BA supplementation. In addition, this study adds BA supplementation as a way to prevent the decline in executive function seen following recovery from endurance exercise.

del Favero *et al* [2] demonstrated an 85% increase in skeletal muscle carnosine content following 12 weeks of BA supplementation 60–80 year old. This study’s use of 2.4 g/day of BA for 28 days to improve skeletal muscle carnosine has been shown to be effective in multiple previous studies and thus was not directly measured in this study [2, 4, 12, 24]. One of our main findings included a BA supplementation directed improvement in TTE during 70% cycle ergometer exercise compared to PL. This confirms the improvements in exercise performance seen in the work from Stout et al [4] who used the same BA supplementation dose and schedule. This small body of work is in agreement to a very recent meta-analysis evaluating effect size of BA supplementation, which stated that BA has its largest impact on extending exercise capacity rather than improving short term performance [30].

Interestingly, despite improved TTE, BA subjects in this study showed no difference in HR, RPE or lactate levels during exercise compared with PL subjects. These findings, consistent with our hypothesis, indicate that BA supplementation is associated with the ability to attenuate physiological stress associated with endurance exercise resulting in delayed fatigue onset.

The second major finding was that BA supplementation mediated the executive function decline that follows transient executive function improvements associated with endurance exercise, a phenomenon reported in recent literature and consistent with our hypothesis. For example, Pollow et al [28] noted that immediately following an endurance exercise bout, Stroop test performance briefly exceeds baseline testing though this effect is transient as it is followed by slowing of executive function [28]. BA’s effects on executive function in the current study are supported by Hoffman et al*’s* [21] ability to detect improved cerebral cortex, hippocampus, amygdala, hypothalamus and thalamus carnosine content through BA supplementation in rodents, as it is thought that improved intracranial carnosine correlates to better executive functioning. However, the results also directly contradict current literature regarding BA’s ability to enhance executive function once utilizing human subjects [9, 19]. Additionally, Solis et al. [22] also investigated BA’s effects on Stroop test performance and saw no benefit with BA supplementation. However, this discrepancy may be due to the fact that the subjects enrolled were already trained cyclists. The differing fitness levels between the current study and that performed by Solis et al. [22] make it difficult to compare results.

Limitations to this study include a sample size of 12. As a result of patient withdrawal and an outlier in the data set, the current study failed to reach its estimated sample size of 13. However, power analysis of the present study’s data revealed a power of 0.9. Secondly, BA’s effect on TTE showed only a moderate effect size of 0.4 despite having a significantly longer POST TTE compared to PRE TTE. This warrants future investigations regarding the relationship between BA supplementation and endurance exercise in the aging population. Thirdly, neither skeletal muscle nor brain carnosine concentrations were assessed, however, the dosing strategy for BA was similar to other work that did see elevations of carnosine in those locations. Finally, while lactate, RPE and HR were measured during and after exercise, blood pH was not. This limited the ability to assess if the improvements in endurance exercise time was due to carnosine directed pH buffering.

## Conclusion

In summary, the current study indicates that 2.4 g/day of BA, given as three, 800 mg doses with a meal per day, for 28 days can improve endurance exercise capacity with no changes in lactate, RPE or HR. This BA dosing strategy was also able to reduce endurance exercise induced declines in executive function. Currently, this is the first investigation of BA supplementation and executive function following recovery from endurance exercise.

## Abbreviations

BA: β-Alanine
GXT: Graded exercise test
HR: Heart rate
PL: Placebo
POST: Post Supplementation
PRE: Pre Supplementation
RPE: Rate of perceived exertion
rpm: Revolutions per minute
TTE: Time-to-exhaustion
W: Watts
